# Malignant paragangliomas with succinate dehydrogenase subunit B mutation in a 13-year old child treated successfully with surgery and 131-I-MIBG

**DOI:** 10.1186/1687-9856-2013-S1-P112

**Published:** 2013-10-03

**Authors:** Chi Kwan Jasmine Chow, PW Yau, CP Wong, Angel Chan, WM But

**Affiliations:** 1 Department of Paediatrics, Queen Elizabeth Hospital Kowloon, Hong Kong; 2 Nuclear Medicine Unit, Queen Elizabeth Hospital, Kowloon, Hong Kong; 3 Department of Pathology, Queen Elizabeth Hospital, Kowloon, Hong Kong

## 

Paragangliomas and phaeochromocytomas are exceptional rare tumours in children arising from the neural crest origin. A number of susceptibility genes have been identified to be associated with familial cases of paragangliomas and phaeochromocytomas. Malignancy frequency has been found to be high especially in patients with succinate dehydrogenase subunit B (SDHB) mutations. We report a case of a 13-year old Chinese girl with right adrenal phaeochromocytoma, paraganglioma in the subhepatic region with invasion into the inferior vena cava, and metastases to the right scapula and vertex. She underwent surgery for excision of the abdominal tumours, and also therapeutic I-131-meta-iodobenzylguanidine (131-I-MIBG) therapy for the metastatic lesions. She required a left adrenalectomy 2 years later due to the occurrence of a left phaeochromocytoma. She remained asymptomatic for 8 years after the initial presentation and is currently alive. Although she does not have any family history of neuroendocrine tumours, susceptibility genes were screened in view of the young age at presentation and multifocal malignant tumours. She was found to carry a known mutation c.572G>A (p.Cys191Tyr) in the *SDHB* gene. This case illustrates the need for screening of susceptibility genes for familial paragangliomas/phaeochromocytomas in apparently sporadic cases, and the therapeutic benefit of using 131-I-MIBG as an adjuvant treatment for metastatic lesions.

